# Instantaneous Self-Powered Sensing System Based on Planar-Structured Rotary Triboelectric Nanogenerator

**DOI:** 10.3390/s21113741

**Published:** 2021-05-28

**Authors:** Shuangyang Kuang, Xiaochen Suo, Peiyi Song, Jianjun Luo

**Affiliations:** 1 CAS Center for Excellence in Nanoscience, Beijing Institute of Nanoenergy and Nanosystems, Chinese Academy of Sciences, Beijing 100083, China; 2018508001@hust.edu.cn; 2 MOE Key Laboratory of Fundamental Physical Quantities Measurement, School of Physics, Huazhong University of Science and Technology, Wuhan 430074, China; d202080048@hust.edu.cn (X.S.); songpeiyi@hust.edu.cn (P.S.); 3 School of Nanoscience and Technology, University of Chinese Academy of Sciences, Beijing 100049, China

**Keywords:** energy harvesting, instantaneous self-powered, triboelectric nanogenerator, RF transmission

## Abstract

Self-powering electronics by harvesting mechanical energy has been widely studied, but most self-powering processes require a long time in the energy harvesting procedure, resulting in low efficiency or even system failure in some specific applications such as instantaneous sensor signal acquisition and transmission. In order to achieve efficient self-powered sensing, we design and construct an instantaneous self-powered sensing system, which puts heavy requirements on generator’s power and power management circuit. Theoretical analysis and experimental results over two types of generators prove that the planar-structured rotary triboelectric nanogenerator possesses many advantages over electromagnetic generator for the circumstances of instantaneous self-powering. In addition, an instantaneous driving mode power management circuit is also introduced showing advanced performance for the instantaneous self-powering sensing system. As a proof-of-concept, an integrated instantaneous self-powered sensing system is demonstrated based on Radio-Frequency transmission. This work demonstrates the potential of instantaneous self-powered sensing systems to be used in a wide range of applications such as smart home, environment monitoring, and security surveillance.

## 1. Introduction

With the development of economics and information technologies, peoples’ demands on personal consumer electronics are becoming larger and broader. The major factor that limits the application and functional evolution of electronics is their dependence on batteries. For most cases, batteries are the guarantee of electronics’ normal functioning but also maybe the trouble under some specific scenarios, such as electricity exhausting in resources limited areas [1] and irreversibly battery aging [2,3]. What is more, to ensure enough electricity, a bulky battery must be carried that severely limits the further reduction of electronics’ size and increases cost, which are harmful to the convenience tendency of electronics. A promising route that makes electronics battery-free is self-powering electronics by converting environmental energy into electricity, such as nanogenerator [4], electromagnetic generator [5], piezoelectric nanogenerator [6], and thermoelectric generator [7]. Among them, triboelectric nanogenerator (TENG) is expected to be an excellent solution due to its high-power density on a per unit volume and mass [8,9]. In recent years, self-powered sensing system based on TENG has been comprehensively studied [10,11], but in most previous studies, a relatively long time is wasted on harvesting mechanical energy before a self-powered sensing process can be successfully deployed [12]. On the one hand, it increases the sensor’s working period and thus causes low working efficiency. On the other hand, it limits applications in some specific scenarios, such as SOS signal acquisition and transmission in emergency disasters [13,14].

In this work, we put forward the concept of the instantaneous self-powered sensing system. With this self-powered sensing system, the sensor’s working does not rely on a battery, and the sensor’s signal can be real-time acquired. It is realized due to two main points. The first point is that the planar-structured rotary TENG (pr-TENG) is selected as the mechanical energy harvester due to its high-power density. The second point is that an instantaneous driving mode power management circuit (PMC) is constructed with a longer duration of discharge current and higher electricity transfer efficiency. In order to put the concept of instantaneous self-powered sensing system into practice, we further design and build an instantaneous self-powered Radio-Frequency (RF) transmission system that is driven by the pr-TENG. The pr-TENG, the instantaneous driving mode PMC, and the RF transmission circuit are integrated into a door shell. Along with an instantaneous period of doorknob rotating by hand, the pr-TENG can harvest the rotational mechanical energy for emitting a self-powered remote-control command. The reported self-powered sensing system in this work shows a wide range of potential applications in smart home, environment monitoring, and security surveillance.

## 2. Design

### 2.1. The Design Considerations for the Instantaneous Self-Powered Sensing System

**Figure 1:**
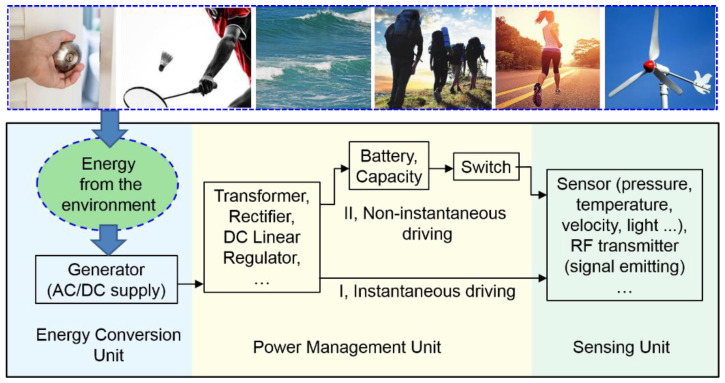
Block diagram of a self-powered sensing system and its working process.

A self-powered sensing system usually consists of three major sub-systems: an energy conversion unit, a power management unit, and a sensing unit, as diagrammed in Figure 1. In the energy conversion unit, environmental mechanical energy or thermal energy is harvested and converted into electricity by the generator. In the power management unit, the unstable output of the generator is regulated to supply stable voltage and current for sensors.

The instantaneous self-powered process means the energy harvesting process and the sensor-powered process shall be simultaneously taken place, and the whole processes are completed in a short period. In order to achieve this goal, two requirements for the system should be met.

The first requirement is that the generator’s output power should be high enough. This is because most electronics’ power is in the range of milliwatt (mW) to watt level. For a generator with a power of 0.1 mW, it takes at least 10 s to harvest energy and complete a successful driving process for the electronics with a power of 1 mW. For these low-power generators, the most common method to realize self-powered mW electronics is to harvest energy in a long period, mostly lasting seconds or even minutes [12,15].

The second requirement is that the power management circuit (PMC) should possess high electricity management efficiency, which means less energy will be wasted. Most previous studies on self-powered electronics divide the self-powered processes into two steps, the energy-harvesting process and the energy-releasing process. The harvested energy is firstly stored in a battery or a capacitor. Then, the stored energy is released to drive electronics when enough energy is harvested. The divided processes are usually controlled by a switch. We call this kind of working process the non-instantaneous driving process because it extends the sensor’s working period to seconds or even minutes, which cannot be considered as an instantaneous action. Conversely, in the instantaneous driving process, the energy-harvesting process and the energy-releasing process occur simultaneously (within one second), which will dramatically decrease the driving period and improve the working efficiency.

In the following text, we will talk about the design of the high-power generator and instantaneous driving mode PMC to meet the requirements for the instantaneous self-powered sensing system.

### 2.2. Design of the High-Power Generator for Instantaneous Driving

Various energy harvesting technologies have been used for building the self-powered sensing system, as shown in Appendix A, Table A1. Among them, the most common generators that possess high-power with mW level are triboelectric nanogenerator [9,16,17] and electromagnetic generator (EMG) [18]. The most promising TENG is the planar-structured rotary TENG (pr-TENG) [9]. The high power of pr-TENG benefits from the high density of periodic grid design. EMG has the same periodic grid design and high power as the pr-TENG. However, as to realize instantaneous driving, pr-TENG and EMG have big differences.

**Figure 2:**
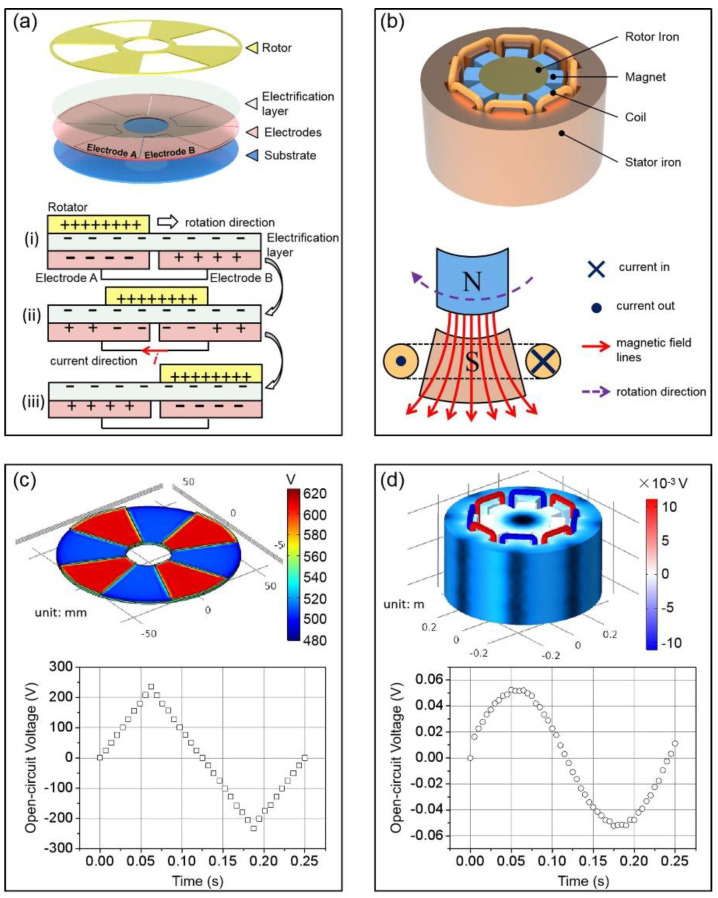
The structural design and COMSOL simulation results of the pr−TENG and EMG. (**a**) Schematic sketch and working mechanism of the pr−TENG; (**b**) schematic sketch and working mechanism of the EMG; (**c**) electrical output of the pr−TENG by COMSOL simulation; (**d**) electrical output of the EMG by COMSOL simulation.

As shown in Figure 2a, the pr-TENG essentially consists of a rotor and a stator. The stator has three components: an electrification layer, an electrode layer, and a substrate. The electrification layer is made of poly tetra fluoroethylene (PTFE) that has the opposite triboelectric polarity against the rotor. The electrode layer is composed of electrode A and electrode B that have complementary planar grids separated by fine gaps. The rotor has the same pattern as one of the electrodes. The rotor and electrode layer are fabricated by the printed circuit board (PCB) manufacturing technology. The key design that makes the pr-TENG possessing high power density is the rotor’s and electrodes’ radial-arrayed periodic grids design. By using the PCB manufacturing technology, the grid number can be increased to hundreds.

When driven by a mechanical force, the rotor spins along the axis in the center. The conversion process of mechanical energy into electricity is illustrated through a structural unit as the rotor spins from position state (i) to state (iii), as shown in the diagram at the bottom of Figure 2a. Under the effect of triboelectrification, the negative charges transfer from the rotor to the electrification layer when the two materials with different triboelectric polarity contact with each other, remaining the positive charges on the rotor. Due to the law of charge conservation, the surface charge density on the rotator is twice as much as that on the stator because the surface area of the rotor is half as much as that of the electrification layer. At state (i), rotor is aligned with electrode A. If the two electrodes are electrically connected, namely, on the short-circuit condition, free charges will redistribute on electrodes due to electrostatic induction: part of negative charges accumulate on electrode A to neutralize with the positive charges on the rotor, and the same quantity of positive charges accumulate on electrode B to neutralize with the negative charges on the adjacent section of the electrification layer. At state (ii), the rotor is situated at the intermediate position of electrodes, and electrons flow from electrode A to electrode B to form a new electrostatic equilibrium. On state (iii), the rotor is aligned with electrode B, exhibiting a position symmetry and charge distribution symmetry with that of state (i). From state (i) to state (iii), electrons flow from electrode A to electrode B to generate the output current.

As another kind of high-power generator, which has been widely used in our daily life, EMG has many similarities comparing to pr-TENG in the device structure, as shown in Figure 2b. The pole N and pole S of the magnet correspond to the rotor with positive triboelectric polarity and electrification layer with negative triboelectric polarity. The radial-arrayed periodic magnet units and coil units correspond to the radial-arrayed periodic rotor grids and electrode grids. The electron flowing in coils corresponds to the electron flowing between electrode A and electrode B.

What is more, the origins of output current in pr-TENG and EMG also have similarities. The open-circuit voltage of EMG *V*_oc_ is proportional to the changing rate of the magnetic flux in each coil loop (dΦ/dt) and the number of total loops (*N*), which can be expressed as below:

(1) $$V_{\mathrm{oc}}=-Nd\Phi /dt$$

Considering the total resistance *R* in EMG, the current in *R* can be expressed as $V_{\mathrm{oc}}/R$, that is,

(2) $$I_{\mathrm{EMG}}\propto d\Phi_{\mathrm{total}}/dt$$

Apparently, the current in *R* is from the total changing rate of the magnetic flux in coils. In TENG, Zhong Lin Wang has initially pointed out that the current in TENG is from the Maxwell displacement current [19], which can be expressed as

(3) $$I_{\mathrm{TENG}}\propto dD_{\mathrm{total}}/dt$$

*D*_total_ is the total electric displacement vector in the space between electrification layers. No matter whether in EMG or in TENG, the changing rate of the magnetic flux or the electric displacement vector is both from the external mechanical force-induced relative position changing between the two sectors with different polarity; one is from the magnet, and the other is from the electret.

The simulation by COMSOL can visualize the similarities above, as shown in Figure 2c,d. The geometric models used in the simulations are the same as models in Figure 2a,b, and parameters of these models can be seen Appendix B. For the pr-TENG, the rotor is set to rotate at a uniform angular speed of 2π/s. The simulated potential distribution and the potential difference between electrode A and electrode B (open-circuit voltage *V*_oc_) are presented in Figure 2c. The peak-to-peak value of the *V*_oc_ curve is about 500 V, which is heavily dependent on the triboelectric charge density on the surface of the electrification layer, and the frequency of the curve is 4 Hz, heavily dependent on the rotation speed and the number (*n*) of grids on the rotor and the electrodes (here *n* = 4). The simulation results of the EMG have similarities but also differences compared with pr-TENG, as presented in Figure 2d. The frequency of the open-circuit voltage is the same as that of pr-TENG because the magnet and coil unit number (*n* = 8) is twice as much as the grid number in pr-TENG, but rotation angular speed is half (here rotation angular speed is π/s). The peak-to-peak value of the curve is only about 0.1 V, much lower than that of pr-TENG. This difference mainly comes from the working mechanism of the two generators; one’s open-circuit voltage is from magnetic flux, and the other is from the electric displacement vector. This feature reveals the excellent advantages of the pr-TENG in harvesting mechanical energy.

## 3. Results and Discussion

### 3.1. Electrical Outputs of Generators

**Figure 3:**
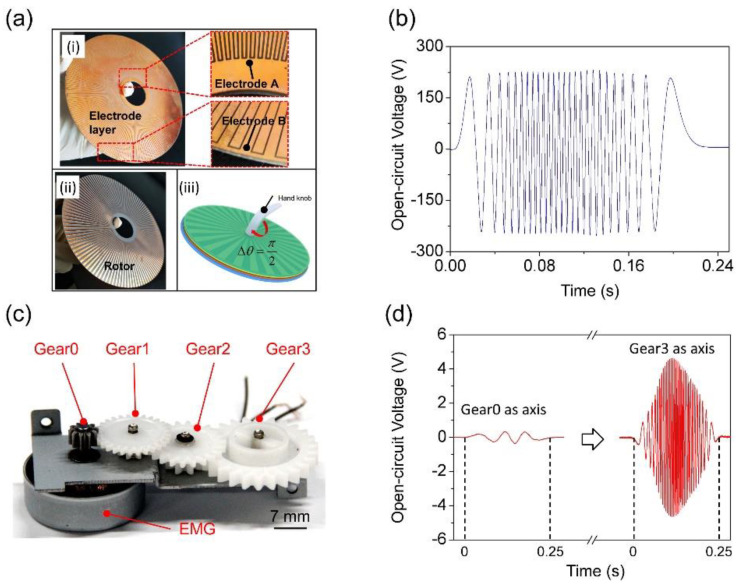
The measured electrical output of pr−TENG and EMG. (**a**) Photographs of the rotor and electrode layer, and the schematic diagram of the pr−TENG; (**b**) open-circuit voltage of the pr−TENG at a rotation speed of 50 rpm; (**c**) photograph of a commercial three-phase alternator for comparison; (**d**) open-circuit voltage of the EMG at a rotation speed of 50 rpm.

To further validate the simulation results, an integrated pr-TENG is fabricated, as presented in Figure 3a. Photographs (i) and (ii) present the electrode layer and the rotor of the pr-TENG. The rotor, electrode A, and electrode B have the same grid number, diameter, and thickness. The grid number is 120. The diameter is 120 mm. The thickness is 0.4 mm. Schematic diagram (iii) presents the assembled pr-TENG. A hand knob is set to be coaxial with the pr-TENG for the purpose of showing the potential application of the pr-TENG in harvesting mechanical energy induced by hand motion like opening a door. The electrical output of the pr-TENG is measured as a slight rotation angle of π/2 occurs at an average rotating speed of approximately 50 rpm.

The *V*_oc_ of pr-TENG is measured by an electrometer (Keithley 6514), as presented in Figure 3b. The *V*_oc_ exhibits a triangular wave with a peak-to-peak value up to 450 V, which is much like the simulation result in Figure 2c. The frequency of *V*_oc_ curve is about 104 Hz, much higher than the simulation result of 4 Hz. This is because the grid number of the real pr-TENG is dramatically increased to 120. It clearly points out an easy way to improve the output power of the pr-TENG, which is to improve the grid’s integration level. The situation is different for the EMG. A commercial three-phase alternator is used for comparison, as presented in Figure 3c, and its detailed size can be seen in Appendix C. The EMG has 4 gears as the rotation axis. The first gear named Gear0 is coaxial with the rotor of EMG. Gears named Gear1 to Gear3 are set to improve the rotation speed of the rotor by the coupling of adjacent gears. The same rotation speed of 50 rpm is employed on Gear0 and Gear3 by a motor. Figure 3d presents the measured *V*_oc_ of the EMG. We can see that when Gear0 is set to be the rotation axis for the EMG, the frequency of *V*_oc_ curve is 12 Hz, and its maximum peak-to-peak value is 0.7 V. When Gear3 is set to be the rotation axis, the frequency of *V*_oc_ curve is 132 Hz. The frequency enhancement is due to the multistep speed changing on the coupling of 4 gears that makes the rotation speed of the rotor increase 11 times; the use of gears to improve rotor’s rotation speed has been previously reported for the TENG [20]. Meanwhile, the maximum peak-to-peak value increases to 9.2 V. Actually, the power of the EMG is usually improved by coupling of multiple gears. Because for the EMG with a confirmed size, the magnet unit number and coil loops usually reach their limits of manufacturing. The only simple and effective method to improve power is to increase the number or transmission ratio of gears. However, this method will bring two adverse effects. First, it will increase the total volume of the generator, which is harmful to the sensing system’s integration. Second, it will increase the damping between two adjacent gears, leading to a decrease in energy conversion efficiency.

The electrical output measurements of the generators validate the simulation results in Figure 2c,d. It shows the performance of high output voltage for the pr-TENG compared with EMG, as having been reported by previous works [9,21]. At the same time, it shows an effective and simple way to improve the output power of the pr-TENG by increasing the grid number on the rotor and electrodes. However, this method is not suitable for EMG. The effective method for the EMG’s output power is to increase the number of gears, but it will bring new problems.

### 3.2. Comparison of the Two Generators in Increasing Their Power

The similar structure and working mechanism of the pr-TENG and EMG make them possess similar routes to improve power. The comparison is summarized in Table 1.

Firstly, the measurements of electrical output in Figure 3 have shown an effective way for improving the power of the pr-TENG by increasing the grid number on rotor and electrodes, which has been reported by Guang Zhu [9]. Changbao Han has made the grid number to be 180 by PCB manufacturing technology [17]. The power of EMG can also be improved by increasing the number of magnet unit. However, due to the size limit of the magnet and coil, their numbers hardly rise to tens or hundreds.

Secondly, increasing the diameter of pr-TENG will dramatically improve its power. This is because the open-circuit voltage of pr-TENG is proportional to the surface area of the electrification layer [22] and absolutely proportional to the square of the diameter. However, increasing radial dimension will decrease the effective magnetic field strength in unit volume for the EMG because effective magnetic field strength only exists in the gap between magnet and iron core, and the useless volume inner the rotor increases quadratically with the increasing of diameter.

Thirdly, increasing the surface charge density on the electrification layer by fluorinated surface modification can improve the power of the pr-TENG [23], which is similar to increasing the magnetic field strength of the magnet for the EMG. However, the magnetic field strength of materials in normal pressure and temperature is hard to improve at present.

Lastly, improving the rotation speed is also an efficient way to improve the generator’s power. It can be achieved by multistep gears’ coupling. It is equivalent for the pr-TENG and EMG in this way.

In summary, from the aspect of improving power, pr-TENG shows excellent advantages in increasing size, improving integration level, and optimizing material’s properties comparing with EMG. In addition, the pr-TENG has advantages of low cost, lightweight, and flexibility [8,24]. These make it much suitable for applications in an instantaneous self-powered sensing system.

### 3.3. Performance of the Instantaneous Driving Mode Power Management Circuit 

Because the operating voltage and current of electronics should be regulated at a stable and safe level, a power management circuit (PMC) is necessary for regulating the electrical output of the generator. As mentioned in Figure 1, one crucial requirement for the instantaneous self-powered sensing system is the PMC with high performance. Here, by comparing two types of PMCs, we point out that the PMC with high performance should possess two main characters.

**Figure 4:**
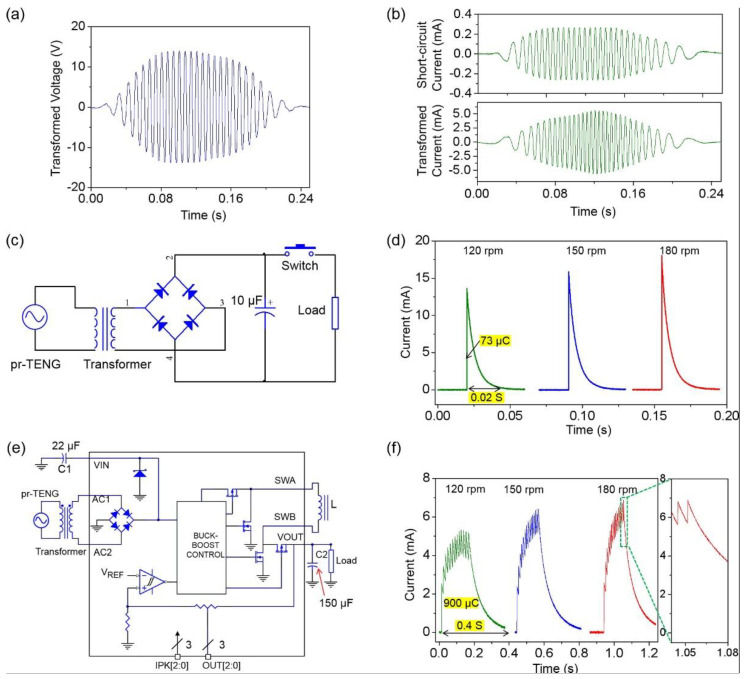
The instantaneous and non-instantaneous driving mode PMCs. (**a**) The transformed voltage of the pr−TENG; (**b**) the short-circuit current and the transformed current of the pr−TENG; (**c**) circuit diagram of the non-instantaneous driving mode PMC; (**d**) driving performance of the non-instantaneous driving mode PMC at different rotation speeds; (**e**) circuit diagram of the instantaneous driving mode PMC; (**f**) driving performance of the instantaneous driving mode PMC at different rotation speeds.

A transformer is first used to reduce the impedance of the pr-TENG to match the impedance of the PMC. With the use of a transformer, the peak-to-peak value of the open-circuit voltage is decreased from 450 to 30 V, as shown in Figure 4a, and the maximum peak-to-peak value of the short-circuit current is enhanced from 0.5 to 10 mA, as shown in Figure 4b. The voltage and current are all measured by an electrometer (Keithley 6514). The first type of PMC is sketched in Figure 4c. The electrical output after transformation is rectified through a full-wave diode bridge, and a 10 μF capacitor is then used as an energy storage unit to reserve the electrical energy. Once the rotor of the pr-TENG finishes a rotation circle by a mechanical driving force, the switch is subsequently turned on. The capacitor then provides sufficient electricity as a power source for a load (510 Ω). The discharge current through the load is first measured to characterize the performance of the PMC. Figure 4d presents the measured discharge current at a fixed rotation angle of π/2 with different rotation speeds (120, 150, and 180 rpm). The measured current is a typical capacitor discharge curve, and the discharge current amplitude increases along with the increasing rotation speed. This is because higher rotation speed brings higher electricity energy density and larger quantity of charges accumulated in the capacitor. It is noted that in our case, the driving processes are divided into two steps by a manual mechanical switch: the energy harvesting process before switch on and the charges releasing process after switch on. As mentioned in Figure 1, this type of divided-working-process PMC is called non-instantaneous driving mode PMC.

As an improvement, the second type of PMC is achieved, as sketched in Figure 4e. A logic chip (LTC3330, Linear Technology) is adopted to substitute the manual mechanical switch in the non-instantaneous driving mode PMC. The LTC3330 integrates a full-wave bridge, a buck-boost converter control chip, a hysteresis comparator, and a buck-boost power switch. It functions as an intelligent switch, which can automatically release stored charges in a capacitor once the voltage of the electricity-stored capacitor reaches a pre-set threshold voltage 5 V; similar works have been previously reported [25,26,27]. The detailed working mechanism can be seen in Appendix D. At the same measurement conditions of the non-instantaneous driving mode PMC, the amplitude of the discharge current through a load increases from 5.5 to 7 mA as the rotation speed varies from 120 to 180 rpm, as shown in Figure 4f. The discharge current is composed of plenty of small peaks, as shown in the magnified inset diagram. This is the result of two processes that occur alternately. As the stored energy in capacitor C2 is being released, the electrical energy is simultaneously replenished from the pr-TENG and stored in capacitor C2. Once the voltage of capacitor C2 reaches a pre-set threshold voltage of 5 V, a new energy-releasing circle begins. The energy-harvesting process and energy-releasing process occur simultaneously. Once the rotation speed increases, the energy-replenishing rate becomes bigger; thus, more charges are accumulated, and the discharge current increases. Once the rotation stops, the energy-replenishing process stops, and the discharge current drops like a capacity discharging process. The energy harvesting-releasing is an alternately succeeding process that apparently differentiates the divided harvesting-releasing process in the non-instantaneous driving mode PMC. This type of PMC is called instantaneous driving mode PMC.

From the measurement results, we can find that the instantaneous driving mode PMC possesses two major benefits compared with the non-instantaneous driving mode PMC. The first is about the electricity transfer efficiency. At the rotation speed of 120 rpm, the accumulated charge in the instantaneous driving mode PMC is about 900 μC, presenting over 12-fold enhancement compared to the non-instantaneous driving mode PMC (73 μC). It indicates the higher electricity transfer efficiency for the instantaneous driving mode PMC. The second is about the long duration of discharge current. At the rotation speed of 120 rpm, the duration of the discharge current is approximately 0.4 s, presenting over 20-fold enhancement compared to the case in the non-instantaneous driving mode PMC (0.02 s). The longer duration time indicates the broader application scenarios for the instantaneous driving mode PMC. Many electronics need long driving times, such as the RF emitter. These two significant benefits indicate that the instantaneous driving mode PMC is more suitable for the instantaneous self-powered sensing system.

### 3.4. Demonstration of an Instantaneous Self-Powered Sensing Application

**Figure 5:**
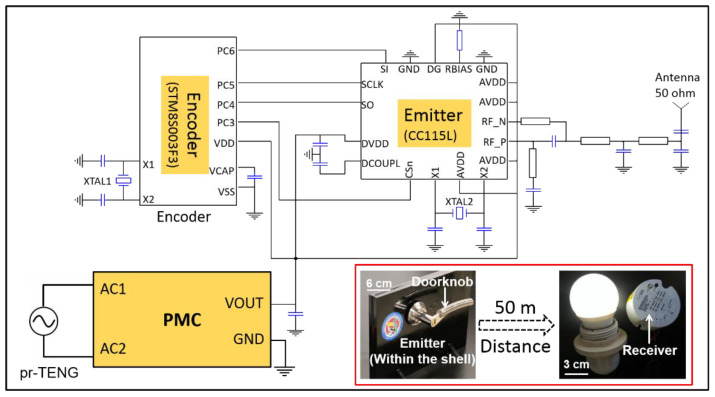
Circuit diagram of the self-powered RF transmission system and the demonstration of remote control.

At last, as a proof-of-concept, an integrated instantaneous self-powered sensing system is constructed. The pr-TENG is selected as the energy harvester, an RF transmission circuit as the sensor which will emit a control command, and the instantaneous driving mode PMC as the energy management circuit to supply stable output for the RF transmission circuit. The detailed system design is presented in Figure 5. The RF transmission circuit is composed of an encoder and an emitter. An 8-bit low-power STM8S003F microcontroller unite (MCU) is used as an encoder to generate a clock/data controlling signal for the emitter. The emitter CC115L is a low-power sub-GHz RF emitter that operates in the frequency ranges of 300–348 MHz, 387–464 MHz, and 779–928 MHz.

This integrated instantaneous self-powered sensing system is constructed as a smart home prototype, in which the pr-TENG, the instantaneous driving mode PMC, and the RF transmission circuit are built in a door shell. The assembly process of the self-powered RF transmission system is shown in Supplementary Content (Figure S1). When the doorknob is rotated, the pr-TENG harvests hand rotational energy and converts it into electricity. The RF transmission circuit is then powered, and it successfully emits a modulated carrier signal. A receiver 60 m away receives the modulated carrier signal (bottom right inset in Figure 5), demodulates the encoding information from the carrier, and executes a command, such as switching on/off a lamp. The whole process is shown in a video in the Supplementary Content (Video S1).

The demonstration shows an instantaneous self-powered data transmission process. The hand-induced mechanical energy that the pr-TENG harvests can act as a power supply for the RF data transmission. The term “instantaneous” here means the energy harvesting and the RF transmission driving are both completed in an instantaneous period (about 0.25 s). Environmental information, such as temperature, humidity, and wind velocity, etc., can be measured by a sensor network, and these data can be transmitted by this instantaneous self-powered sensing system for remote control applications.

## 4. Conclusions

To summarize, in this work, we construct an instantaneous self-powered sensing system. For this purpose, we first point out that two requirements should be met, one is the generator’s output power should be high enough, the other is the power management circuit should possess high electricity management efficiency. For the first requirement, we introduce the pr-TENG and EMG as the high-power energy harvesters and demonstrate that the pr-TENG possesses significant advantages in improving power compared to EMG, such as hundreds of grids design. Taking other advantages into consideration, we conclude that pr-TENG is very suitable for instantaneous self-powered sensing systems. For the second requirement, we design an instantaneous driving mode PMC based on logic chip LTC3330. It presents a longer duration of discharge current and higher electricity transfer efficiency. At last, we design a real instantaneous self-powered sensing system based on the planar-structured rotary TENG and successfully achieve instantaneous RF transmission self-powered by the TENG. Meanwhile, we construct a smart home prototype based on the self-powered RF transmission system. The pr-TENG, the instantaneous driving mode PMC, and the RF transmission circuit are integrated into a door shell. Along with an instantaneous period of doorknob rotating by hand, the pr-TENG harvests the rotational mechanical energy, and a self-powered remote-control command is subsequently emitted. Considering that the transmitter (CC115L) is the main load of the instantaneous self-powered RF transmission system and the current consumption in the transmitter varies from nA to mA for different operation modes, we will take further steps to achieve an efficient instantaneous self-powered RF transmission system in our following studies, such as optimizing the instantaneous driving mode PMC to achieve an efficient way to operate the transmitter. This work not only demonstrates a clear route to achieve higher working efficiency and a shorter working period for the self-powered sensing system but also explores a broader range of potential applications in the smart home, environment monitoring, and security surveillance.

## Figures and Tables

**Table 1:** Routes of improving generators’ power.

Routes	Planar-Structured Rotary TENG	Electromagnetic Generator
integration	increase grid number	increase turn number of coil
size	increase size in radial dimension	increase size in radial dimension
material	increase surface charge density of electrification layers	increase magnetic field intensity of the magnet
rotation speed	increase rotation speed	increase rotation speed

## Data Availability

Not applicable.

## Supplementary Materials

The following are available online at https://www.mdpi.com/article/10.3390/s21113741/s1, Figure S1: The assembly process of the self-powered RF transmission system.

## Appendix A

**Table A1:** The comparison of different environmental energy harvesting technologies in self-powered sensing system applications.

Environmental Energy Harvesting Technology	Advantages	Disadvantages	Applications in Self-Powered Sensing System
Triboelectric nanogenerator based	high power; great advantage in low-frequency mechanical energy harvesting; low cost; lightweight; flexibility	energy loss induced by friction damping; difficulty in power management circuit design	environmental monitoring [28]; self-powered pressure sensor [29]; data acquisition/transmission [11]
Electromagnetic generator based	high power; great advantage in high-frequency mechanical energy harvesting	bulky; hard to integrate with electronics	vibration monitoring [30]; traffic monitoring [31]
Piezoelectric nanogenerator based	high output voltage; light weight; high integration level	fragile; low power	data transmission [6]; self-powered pressure sensor [32]
Thermoelectric generator based	thermal energy harvesting; flexibility; wearable	low power; not applicable in mechanical energy harvesting	health monitoring [33]

## Appendix B

COMSOL simulation of the pr-TENG: the rotor and the stator have the same diameter of 120 mm; the thickness of rotor, electrification layer, and electrodes is 1 mm; the grid number on the rotor, electrode A and electrode B are 4; the surface charge density on electrification layer is −0.73 μC/m^2^.

COMSOL simulation of EMG: the rotor’s diameter is 430 mm; the stator’s outer diameter is 800 mm, inner diameter is 470 mm; the thickness of rotor and stator is 400 mm; the magnet unit number and coils number are 8; the loop number in one coil is 1000; the magnet’s electrical conductivity is 10 S/m, relative magnetic conductivity is 1.05, the residual magnetic flux density is 1.4 T.

## Appendix C

The EMG we used in our experiments is a commercial three-phase alternator, as shown in Figure 3c in the main text. The total size of EMG (including gears) is 70 mm × 30 mm × 20 mm. The EMG’s diameter is 28 mm, and the thickness is 13 mm. The magnet unit number and coil number are 9. It has 4 gears for speed change. The gears’ diameter from Gear0 to Gear3 is successively 7 mm, 18 mm, 19 mm, and 28 mm. The transmission ratio between adjacent gears is successively 18:7, 19:8, 28:10, thus if the rotation speed of Gear4 is 50 rpm, the rotation speed of the rotor will be 855 rpm.

## Appendix D

As shown in Figure 4e in the main text, the LTC3330 integrates a full-wave bridge, a buck-boost converter control chip, a hysteresis comparator, and a buck-boost power switch. The alternating output from pr-TENG is transformed by a transformer and rectified by the full-wave bridge, and then stored in the capacitor C1. The hysteresis comparator monitors the voltage of the capacitor C2 in real-time. When the voltage of capacitor C2 is below 5 V, the comparator produces a low-level output, making the buck-boost power switch at the buck mode. At this mode, the control chip, the buck-boost power switch, and the capacitors C1/C2 make up a complete buck circuit. The electricity stored in capacitor C1 is transferred into capacitor C2. As a result, the voltage of capacitor C2 increases to 5 V. The comparator then produces a high-level output, making the control chip change the buck-boost power switch to discharging mode immediately. At this mode, the buck circuit is disconnected, the capacitor C2 acts as a power source for the load. Another storage-release cycle begins once the voltage of capacitor C2 drops.
