# Supplementary material for: Instantaneous Self-Powered Sensing System Based on Planar-Structured Rotary Triboelectric Nanogenerator
Source: Sensors (Basel). 2021 May 28;21(11):3741. doi: 10.3390/s21113741 (PMC8197852; doi:10.3390/s21113741)
Supplement: Supplementary file 1 [file sensors-21-03741-s001.zip › sensors-1214381-supplementary.pdf]

sensors

MDPI

Article

Instantaneous Self-Powered Sensing System Based on Planar-Structured Rotary Triboelectric Nanogenerator

Shuangyang Kuang [1,2,3], Xiaochen Suo [2], Peiyi Song [2] and Jianjun Luo [1,3,*]

[1] CAS Center for Excellence in Nanoscience, Beijing Institute of Nanoenergy and Nanosystems, Chinese Academy of Sciences, Beijing 100083, China
[2] MOE Key Laboratory of Fundamental Physical Quantities Measurement, School of Physics, Huazhong University of Science and Technology, Wuhan 430074, China; 2018508001@hust.edu.cn
[3] School of Nanoscience and Technology, University of Chinese Academy of Sciences, Beijing 100049, China
* Correspondence: Correspondence: luojianjun@binn.cas.cn (J.L.)

The assembly process of the self-powered RF transmission system in the door shell is shown in the following figure: make two door shells; make the stator by fixing the electrode layer on a substrate made of PMMA, and fixing a layer of PTFE on the electrode layer; fix the stator on one of the door shells; fix the transformer, the PMC, and the emitter module on the door shell; connect the pr-TENG, the transformer, the PMC, and the emitter module; fix the bought doorknob on another door shell; fix the rotor substrate on the doorknob; fix the rotor on the rotor substrate; fasten the two door shells together, and make the rotor and stator to be coaxial.

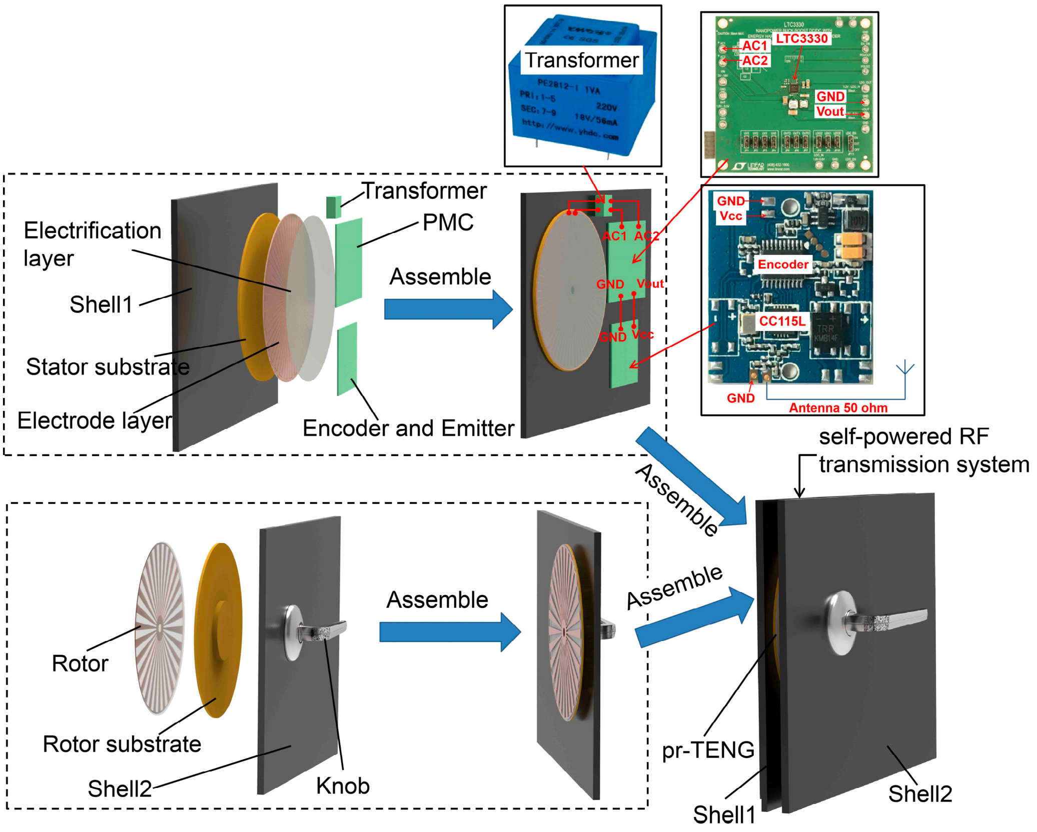

Figure S1. The assembly process of the self-powered RF transmission system.
